# Tracheobronchitis in ulcerative colitis: a case report of therapeutic response with infliximab and review of the literature

**DOI:** 10.1186/s12876-019-1091-0

**Published:** 2019-11-01

**Authors:** Lisa Horgan, Siobhain Mulrennan, Lloyd D’Orsogna, Andrew McLean-Tooke

**Affiliations:** 1 Department of Immunology, Sir Charles Gairdner Hospital, Perth, WA 6009 Australia; 2 Department of Respiratory Medicine, Sir Charles Gairdner Hospital, Perth, WA 6009 Australia; 3 Faculty of Health and Medical Sciences, University of Western Australia, Perth, WA 6009 Australia; 4 Department of Immunology, Fiona Stanley Hospital, Perth, Australia; 5 Pathwest, QEII, Perth, Nedlands Australia

**Keywords:** Ulcerative colitis, Inflammatory bowel disease, Tracheobronchitis

## Abstract

**Background:**

The extra-intestinal manifestation of tracheobronchitis is a rare complication of ulcerative colitis (UC). Here, we present a case of UC-related tracheobronchitis wherein the positive clinical effects of infliximab are demonstrated.

**Case presentation:**

We report the case of a 39-year old woman who presented with a chronic productive cough on a distant background of surgically managed ulcerative colitis (UC). Our patient failed to achieve a satisfactory clinical improvement despite treatment with high dose inhaled corticosteroids, oral corticosteroids and azathioprine. Infliximab therapy was commenced and was demonstrated to achieve macroscopic and symptomatic remission of disease.

**Conclusions:**

We present the first case report documenting the benefits of infliximab in UC-related tracheobronchitis.

## Background

Extra-intestinal manifestations of inflammatory bowel disease (IBD) are well recognised and an association between pulmonary disease and inflammatory bowel disease was first proposed about 40 years ago [1]. Clinically apparent IBD-related pulmonary disease is rare, although the described prevalence increases considerably with inclusion of subclinical lung involvement estimated to be present in 40–60% of the IBD patient cohort [2–5].Pulmonary manifestations of IBD are more commonly found in combination with ulcerative colitis than Crohn’s disease [2] and have a tendency to involve the large airways inclusive of tracheobronchitis, bronchiolitis and bronchiectasis [6]. Ulcerative colitis-related tracheobronchitis is an often an under-recognised entity [7] requiring a high index of suspicion. This is further exacerbated by the tendency towards a delayed presentation and many present over 20 years after the initial diagnosis of IBD [8]. Respiratory symptoms may coincide with the first presentation of UC, but equally may precede it or occur many years after colectomy [7, 9, 10]. Exacerbations of respiratory symptoms do not typically correlate with flares of IBD and may occur during periods of otherwise quiescent disease [9, 11]. It has been proposed that a recent colectomy may have a causal relationship with the progression of respiratory symptoms [12], possibly due the common embryonic ancestry of the bowel and the tracheobronchial tree [13]. Lung manifestations of ulcerative colitis are variable with reported involvement of the upper and lower airway, small airways and lung parenchyma. Many cases (30%) relate to tracheobronchial involvement inclusive of bronchiectasis, bronchitis, tracheobronchitis and bronchiolitis [2].

Oral and inhaled steroids have been the mainstay of the treatment of pulmonary manifestations in UC used in about 65% of cases [2]. Nonetheless, 12–30% of patients relapse on weaning or cessation of corticosteroids requiring re-commencement or up-titration of dose [14]. Steroid-sparing agents such as azathioprine have proven efficacious in achieving disease remission although the data is limited and based mainly on case reports [7, 9, 15]. Infliximab, a chimeric anti-tumour necrosis factor (TNF) monoclonal antibody, has demonstrated promising results from limited numbers of case reports describing its usage in cases of Crohn’s Disease (CD) -related pulmonary disease [16–18]. Despite this experiential evidence for the use of infliximab in lung manifestations of UC has not yet been described in the literature. We report a unique case of delayed-onset tracheobronchitis in a female patient with long-standing surgically managed UC. The approach to reaching the diagnosis of UC-related tracheobronchitis and the approach to management are outlined providing context. Additionally, a review of the literature highlights the potential for pulmonary manifestations of UC to have a delayed presentation inclusive of post-colectomy. The utility of infliximab is emphasised as a therapeutic avenue in the setting of UC-related tracheobronchitis proving refractory to standard treatment with high-dose inhaled or systemic corticosteroids. To our knowledge, this will be the first case report documenting the use of infliximab in UC-related tracheobronchitis hence contributing significantly to existing experiential evidence of this condition.

## Case presentation

**Fig. 1:**
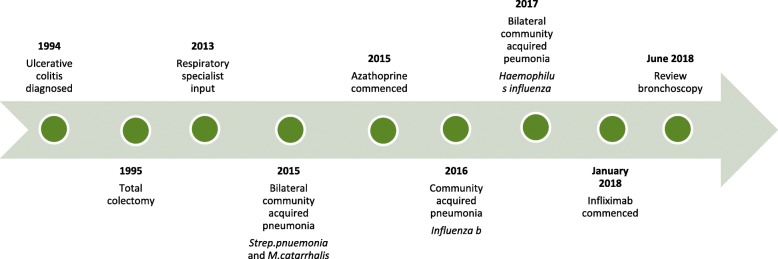
Timeline of Clinical History. Timeline of clinical history

A 34-year-old woman was referred with a persistent productive cough despite completing empirical treatment for presumed infective aetiologies with multiple-broad spectrum antibiotics. As outlined in Fig. 1, this occurred on a background of ulcerative colitis diagnosed in 1994 (age 15 years) and progressing to surgical management of a panproctocolectomy with subsequent J-Pouch formation in 1995.

**Fig. 2:**
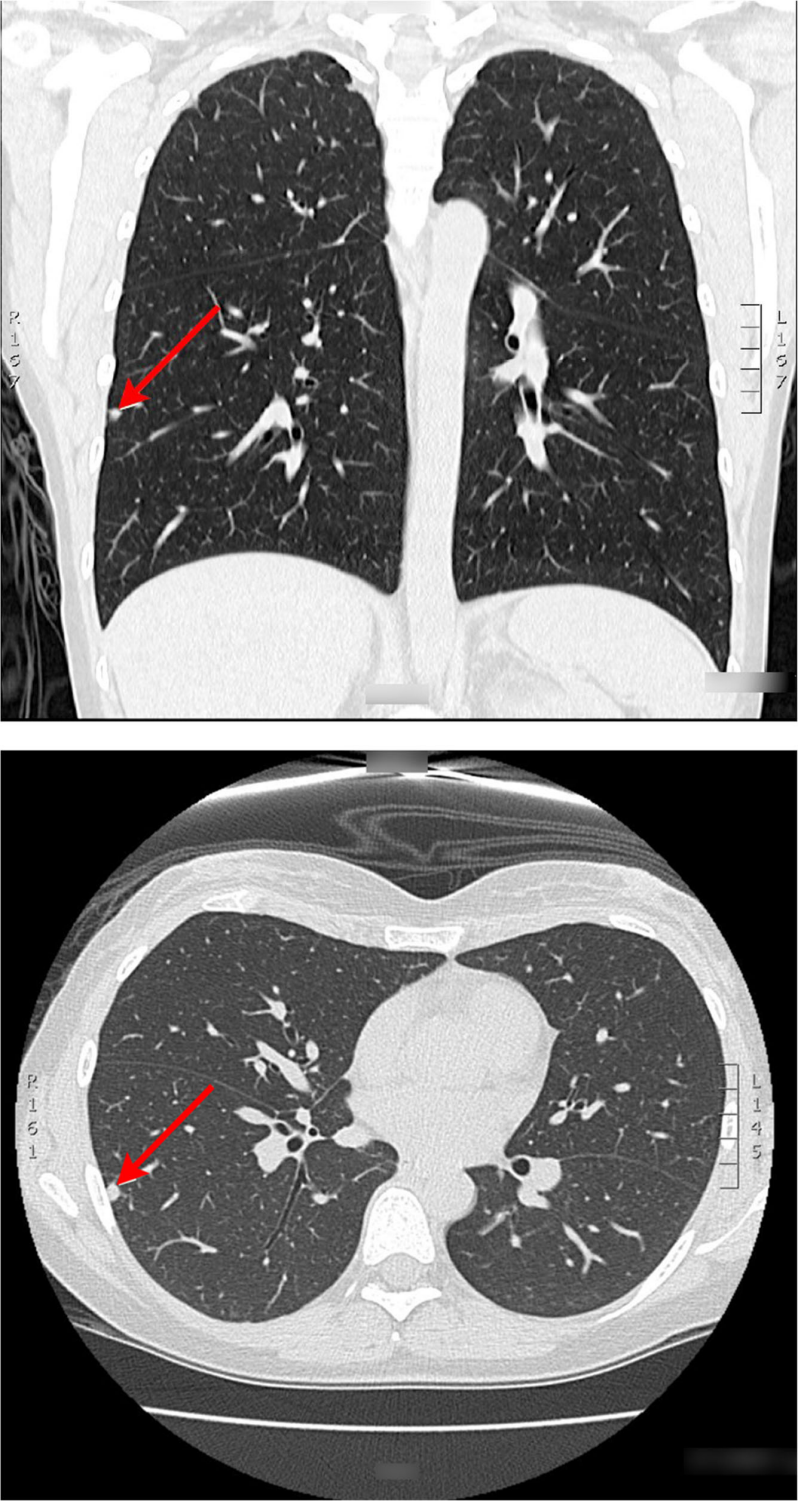
Computed Tomography of Chest, 2013. CT of chest performed in 2013 on first referral to respiratory specialist. CT demonstrates the transient finding of a 6 mm right lower lobe sub pleural nodule, which was transient on serial imaging

**Fig. 3:**
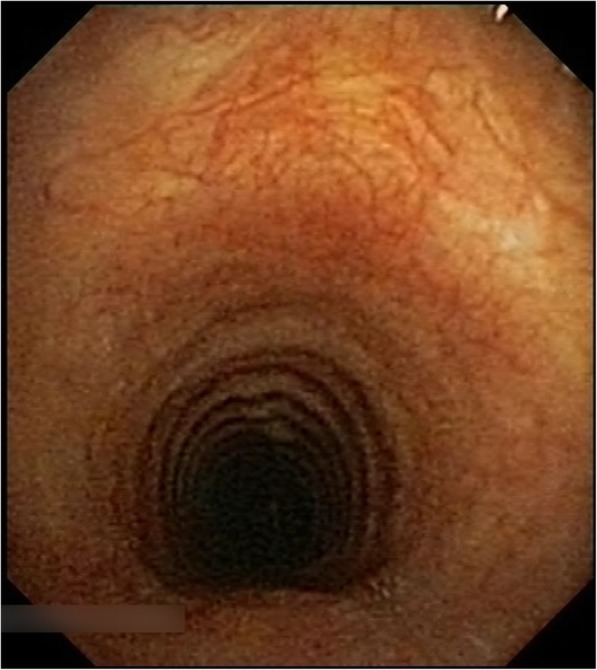
Bronchoscopy, 2016. Image from bronchoscopy performed in 2016 which demonstrates macroscopic evidence of tracheobronchitis despite treatment with azathioprine and high-dose inhaled corticosteroids

Examination did not reveal signs of upper or lower airways disease. Initial laboratory investigations demonstrated the following: white cell count 6.79 × 10^9^/L (neutrophils 5.99 × 10^9^/L, lymphocytes 0.47 × 10^9^/L), C-reactive protein 20 mg/L (normal < 0.5 mg/L), IgG 13.3 g/L (normal 5.8–13.7 g/L), IgM 0.73 g/L (normal 0.30–1.70 g/L). Subsequent serology demonstrated the presence of anti-neutrophil cytoplasmic antibodies (ANCA) but without PR3 and MPO reactivity. Anti-nuclear antibodies (ANA) were not present. Sputum cultures grew *Moraxella catarrhalis* transiently in 2013 with no fungi or acid fast bacilli noted. Pulmonary function testing revealed maximum expiratory flow rates, normal lung volumes and gas transfer resulted as (%predicted) FVC 108%, FEV1 99%, FEV1/FVC 90%, TLC 111%, DLCO corrected 98%. A computed tomography of chest (Fig. 2) revealed a 6 mm right lower lobe sub pleural nodule, which was transient on serial imaging. Initial bronchoscopy was performed prior to our review and showed macroscopic evidence of tracheobronchitis with granularity and purulent inflammation of the bronchial mucosa in the trachea, central and proximal cartilaginous airways with normal appearance of the sub-segmental airways. Histology of bronchoscopy samples showed a dense infiltration of a mixed inflammatory cell infiltrate with relative absence of eosinophil was reported on histopathology review of bronchoscopy specimens. A further surveillance bronchoscopy performed in 2016 (Fig. 3) showed persistent evidence of tracheobronchitis despite patient adherence to a treatment regimen of azathioprine and high dose inhaled corticosteroids.

**Fig. 4:**
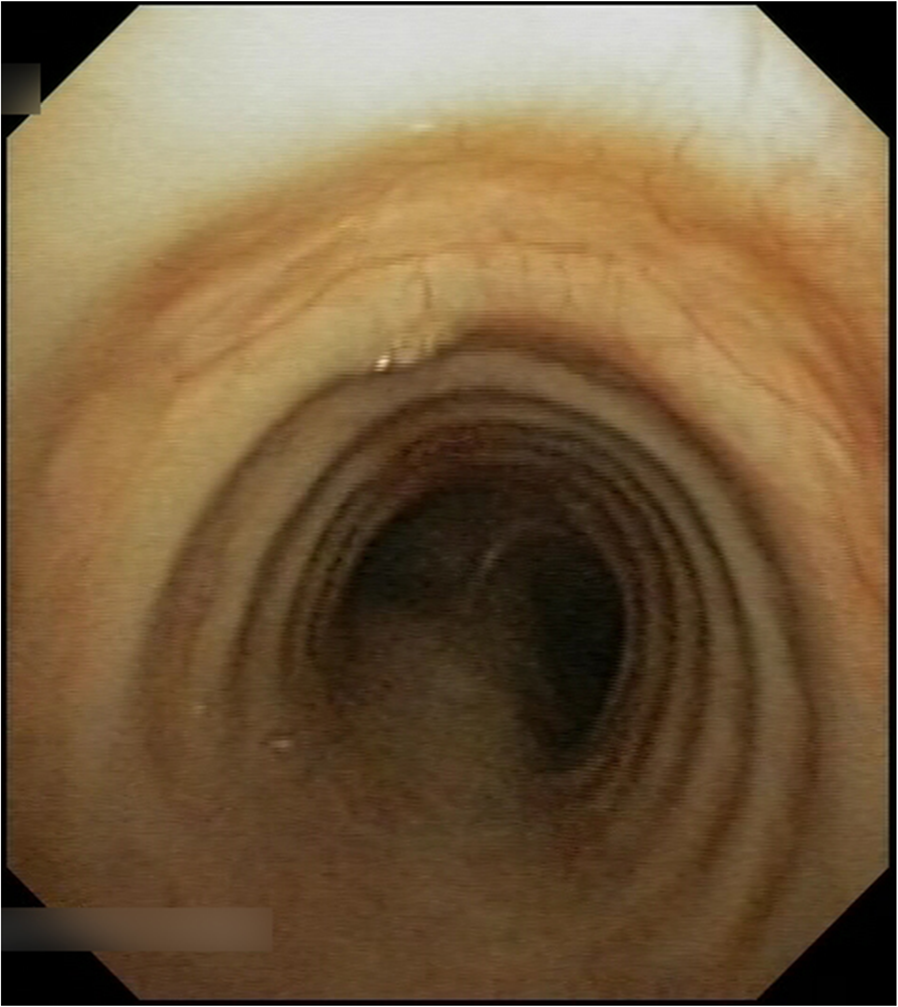
Bronchoscopy, 2018. Image from bronchoscopy performed in 2018 and 5 months post commencement of infliximab. This image demonstrates no macroscopic evidence of mucosal irregularities such as oedema, hyperaemia or ulceration to suggest ongoing active tracheobronchitis following the commencement of infliximab

The patient demonstrated reproducible clinical improvement with oral prednisolone (dose range 0-50 mg daily) and typical symptom relapse on weaning/cessation. This occurred most dramatically in 2015 following a period of management with inhaled high-dose steroids alone and culminated in an acute admission with a *Streptococcus pneumonae* and *Moraxella catarrhalis* generated community acquired bilateral lobar pneumonia with type 1 respiratory failure and an exacerbation of tracheobronchitis. Azathioprine was commenced as a steroid sparing agent in late 2015. Repeat bronchoscopy following 4 months of commencement demonstrated persistent low-grade tracheobronchitis despite treatment with azathioprine (100 mg/day) and inhaled fluticasone propionate (3 g/day). A further tracheobronchitis decompensation driven by respiratory syncytial virus (RSV) resulted in an inpatient admission in early- 2017. Subsequently a further exacerbation caused by *Haemophilus influenzae* in mid-2017 resulted in type 1 respiratory failure requiring high acuity inpatient care. Given the burden of disease despite azathioprine, oral prednisolone and high-dose inhaled corticosteroids, Infliximab induction therapy (0, 2, 6 weeks) and subsequent maintenance therapy (8 weekly) at dose 5 mg/kg was commenced in early 2018 in consideration of refractory symptoms requiring chronic use of oral prednisolone despite the combined treatment regimen of high-dose inhaled corticosteroids and azathioprine. Access to Infliximab was funded by the treating facility. Appropriate pre-screening for latent tuberculosis, varicella zoster virus and hepatitis B virus was undertaken. Infusions of infliximab were well tolerated by the patient with no acute or delayed infusion-related infliximab reactions experienced. Repeat bronchoscopy (Fig. 4) performed approximately 5- months’ post commencement of Infliximab revealed no macroscopic evidence of mucosal irregularities such as oedema, hyperaemia or ulceration to suggest ongoing active tracheobronchitis with an absence of the purulent secretions present on prior bronchoscopy. Infliximab therapy has allowed cessation of oral prednisolone, the gradual weaning regimen of inhaled fluticasone propionate to 1000mcg daily. Clinical stability of the patient has also been achieved with no further exacerbations of tracheobronchitis since commencement of Infliximab and resolution of her productive cough and dyspnoea. As such, maintenance Infliximab therapy has continued in combination with ongoing 3 monthly specialist reviews.

## Discussion and conclusions

IBD-related pulmonary disease remains a relatively rare but well described complication of IBD [2, 14]. UC involvement within the tracheobronchial tree is diverse and may manifest as subglottic stenosis, tracheitis, tracheobronchitis, chronic bronchitis or bronchiolitis [2]. UC involvement of the lung parenchyma presents predominantly as bronchiolitis obliterans organising pneumonia (BOOP) or interstitial lung disease with varying pathological patterns [2]. Large airway involvement typically manifests as productive or non-productive cough, dyspnoea, wheeze or a decline in exercise tolerance. The natural history of symptomatic tracheobronchitis includes progression to irreversible airway stenosis, obliteration of airways and potentially respiratory failure [19, 20].

Tracheobronchitis, inflammation of the trachea and bronchi, may develop at any point during the trajectory of UC. This is supported by a review of 15 reported cases of UC related tracheobronchitis [10] wherein the onset of symptomatic tracheobronchitis varied from the same time as the diagnosis to distantly from intestinal disease activity. The onset of our patient’s respiratory symptoms, occurring more than a decade post-colectomy, correlates with cases outlined in Table 1.

**Table 1:** A Description of Patients with UC-related Tracheobronchitis Occurring Late after Colectomy

	Vashista S et al. [21]	Vashista S et al. [21]	Wilcox et al. [19]	Garg K et al. [22]	Ocak I et al. [11]
Sex	Male	Male	Male	Female	Male
Age at diagnosis of UC (years)	28	59	34	13	18
Age at colectomy (years)	42	59	35	28	40
Duration of UC at onset of tracheobronchitis symptoms (years)	16	5	16	27	35
Years since colectomy	2	5	15	12	13
Treatment	High dose inhaled beclomethasone Prednisolone 5-15 mg daily	High dose inhaled beclo-methasone Prednisolone (unspecified dose)	Manual dilation of distal trachea and main bronchi at rigid bronchoscope Prednisolone 20 mg/day	Not specified	Prednisolone 20 mg/day
Outcome	Improved symptoms	Improved symptoms	Transient improvement of symptoms Iatrogenic ruputure of bronchus during bronchus redilation procedure.	Not specified	Improved symptoms

UC-related pulmonary disease can pose a diagnostic challenge particularly when the remission of intestinal disease is considered falsely reassuring, and not given due consideration. Infectious aetiology is typically a strong differential arising from exposure to various forms of immunosuppression. Although our patient was appropriately screened for infection, the absence of significant immunosuppression preceding or at the onset of respiratory symptoms made this differential less likely. Our patient was infected at various times with M.catarrhalis and H. influenza both exacerbating pre-existing tracheobronchitis rather than instigating. Autoimmune aetiologies including granulomatosis with polyangiitis, sarcoidosis and rheumatoid arthritis where considered and subsequently refuted by the absence of supportive serology. The presence of a normal total IgG excluded common variable immunodeficiency (CVID), which may be associated with IBD and unlike the majority of other primary immunodeficiency syndromes, can have an onset in adulthood [23]. Our patient did not have a smoking history or occupational history to support the differential of irritant-related tracheobronchitis.

Both normal and obstructive pattern pulmonary function testing have been reported in cases of symptomatic IBD-related tracheobronchitis [10, 14, 24]. Our patient had normal spirometry results despite significant respiratory symptoms. Our patient did not progress to methacholine testing; negative reversibility testing can be instrumental in excluding the differential of asthma. Radiological findings of IBD-related tracheobronchitis have included the variable presence of thickening of the bronchial and trachea wall [24–26] reinforcing the diagnostic benefit of bronchoscopy. In keeping with previously recorded cases of tracheobronchitis in IBD [14, 20, 27] our patient had evidence of mucosal oedema and hyperaemia on bronchoscopy. In addition, the dominant neutrophilia noted in bronchoalveolar lavage (BAL) fluid prior to infliximab treatment is a described feature of IBD-related tracheobronchitis.^16^ Bronchial biopsy typically reveals a mixed cellular infiltration including lymphocytes, plasma cells and an absence of granulomas [9, 20].

Treatment regimens for UC-related tracheobronchitis have relied heavily on inhaled, oral and intravenous corticosteroids. The predictable and timely clinical improvement with steroids serves as a strong diagnostic indicator of UC-related pulmonary disease. As outlined in Table 1, the majority of cases received oral corticosteroids and some additionally received high-dose inhaled beclomethasone. Azathioprine has historically been the steroid-sparing agent of choice for pulmonary IBD and is supported by clinical improvement described in case reports [7, 28]. Our patient failed to achieve complete remission following the commencement of Azathioprine either clinically or histologically as demonstrated by a persistent neutrophil and macrophage infiltration evident on bronchoalveolar lavage (BAL) fluid. Additionally, the intended steroid-sparing effect of Azathioprine was not achieved; our patient relapsed following the gradual cessation of oral corticosteroids despite the continuation of high-dose inhaled corticosteroids.

Infliximab, a chimeric anti-tumour necrosis factor (TNF) monoclonal antibody, is established as a rescue therapy in cases of acute severe steroid refractory intestinal ulcerative colitis [29]. Activated lymphocytes, macrophages and other cells express the transmembrane form of TNF-alpha which is processed by the TNF-alpha conversion enzyme (TACE) to generate soluble TNF. Through binding to the receptors for TNF (TNFR1 and TNFRII) soluble TNF promotes mucosal inflammation through various described mechanisms including the destruction of the intestinal barrier, secretion of cytokines and chemokines from intestinal epithelial and apoptosis of epithelial cells [30]. Significantly greater serum and mucosal TNF levels are reported in acute relapse of IBD and supports current opinion that TNF production contributes to the pathophysiology of both ulcerative colitis and Crohn’s disease [31–34]. Infliximab binds both the soluble and transmembrane forms of TNF-alpha with high affinity, blocking their action and promoting mucosal healing [35], leukocyte apoptosis [36] and clinical remission. Although not clearly defined in the literature, it is probable that the mechanism of action for infliximab in pulmonary IBD is similar. Infliximab is described in the literature as a management strategy for pulmonary CD [16, 17, 37, 38] including the promising outcome of disease remission.

There is a lack of published evidence documenting the use of infliximab in pulmonary UC. As such, the use of Infliximab in pulmonary IBD is guided only by sporadic case reports of pulmonary Crohn’s disease [16–18, 37–40] only one of which [40] relates to the diagnosis of tracheobronchitis. Clinical and radiological remission was achieved in each of the 7 described cases commenced on infliximab. The decision to commence infliximab was in the setting of previously refractory disease either failing to respond adequately to or intolerant of systemic corticosteroids and/or azathioprine. Response to infliximab therapy was assessed in all described cases with a combination of radiological imaging and clinical assessment. Of the existing cases described in the literature the duration of therapy varied however with the majority established on maintenance infliximab therapy at the time of case reporting.

This novel case report describes the first trial of infliximab or an anti-TNF monoclonal antibody for the rare and potentially life-threatening extra-intestinal manifestation of UC-related tracheobronchitis. This study further contributes to the literature regarding pulmonary IBD and emphasises the need for a high level of suspicion when assessing a patient with respiratory complaints on a background of IBD regardless of the activity of intestinal disease. Moreover, this unique case provides valuable experiential support for a trial of infliximab in cases of refractory UC-related tracheobronchitis and supports consideration of a more aggressive management approach in such challenging cases.

## Data Availability

Data supporting our case presentation can be found in clinical documentation pertaining to patient’s clinical reviews by treating specialists (in inpatient and outpatient clinical settings), imaging reports (sourced from IMPAX database), pathology results reported by Pathwest laboratories, Perth, WA. All data generated or analysed during this study are included in this published article [and its supplementary information files].

## Abbreviations

ANA: Anti-nuclear antibodies
ANCA: Anti-neutrophil cytoplasmic antibodies
BAL: Bronchoalveolar lavage
BOOP: Bronchiolitis obliterans organising pneumonia
CD: Crohn’s disease
IBD: Inflammatory bowel disease
RSV: Respiratory syncytial virus
TNF: Tumour necrosis factor
UC: Ulcerative colitis
